# Plates Discolored in Repairing

**Published:** 1864-02

**Authors:** A. Berry


### PLATES DISCOLORED IN REPAIRING.

BY A. BERRY, D. D. S.

In Dr. Talbert's communication in the last Register is the parenthetic remark, that he wished somebody would tell him how to remove the black deposit on plates after heating to repair them.

In my dental reading I do not recollect to have seen any thing on this subject previously.

Attached very firmly to the plates, not acted on by acids, usually found in greatest quantity in depressions, about clasps, under and around the teeth, where it is difficult to act on it mechanically, the removal of this black substance involves much trouble.

During several years, pieces that came into my hands for repairs were boiled in wood ashes and water, (having been told it would lessen the liability of breaking the teeth in soldering,) and I think the black deposit was not present on any plates so treated. They were enveloped in the ashes in a ladle, water put in to rise above the ashes, and heated to a red heat. In re-soldering, the teeth seemed not to break so much as when invested as received from the patient. At least there was the advantage of breaking them generally before putting them under the blowpipe.

After abandoning this preparatory plan, I usually washed the pieces to be repaired, and heated them quite hot by a fire or in a stove, before investing in plaster; but on heating they generally had the troublesome deposit on them, although in less quantity than when cleansing was neglected before the investment. Probably boiling the plates a few minutes in an alkaline solution would remedy the difficulty.

To remove the black substance, an active mechanical agent is required. I commonly use pumice stone with pine sticks.

By the way, will not some one give in the Register a mode of treatment to avoid breaking teeth when heating them for repairs?

---

Dr. Clippinger says:—Tell Dr. Talbert to soak his gold plates for a couple of minutes in sulphuric acid before investing them to solder them. Then when he puts them in the acid to pickle or to remove the film, it will remove the other also, the heat and acid having destroyed it.—Ed.

